# Pressure Induced Liquid-to-Liquid Transition in Zr-based Supercooled Melts and Pressure Quenched Glasses

**DOI:** 10.1038/s41598-017-06890-w

**Published:** 2017-07-26

**Authors:** W. Dmowski, S. Gierlotka, Z. Wang, Y. Yokoyama, B. Palosz, T. Egami

**Affiliations:** 1 Department of Materials Science and Engineering, University of Tennessee, Knoxville, TN 37996 USA; 2 Institute of High Pressure Physics, Polish Academy of Science, Warsaw, Poland; 3 Materials Research Institute, Tohoku University, Sendai, Japan; 4 Oak Ridge National Laboratory, Oak Ridge, TN 37831 USA

## Abstract

Through high-energy x-ray diffraction and atomic pair density function analysis we find that Zr-based metallic alloy, heated to the supercooled liquid state under hydrostatic pressure and then quenched to room temperature, exhibits a distinct glassy structure. The PDF indicates that the Zr-Zr distances in this glass are significantly reduced compared to those quenched without pressure. Annealing at the glass transition temperature at ambient pressure reverses structural changes and the initial glassy state is recovered. This result suggests that pressure causes a liquid-to-liquid phase transition in this metallic alloy supercooled melt. Such a pressure induced transition is known for covalent liquids, but has not been observed for metallic liquids. The High Pressure Quenched glasses are stable in ambient conditions after decompression.

## Introduction

In crystalline solids changes in temperature or pressure can induce a phase transition from one structure to another with the same chemical composition (polymorphs). Similar transitions can occur in liquids and glasses, and they are termed “polyamorphic” transition^1, 2^. “Polyamorphic” transition induced by pressure was first observed for the Ce_55_Al_45_ metallic glasses at room temperature (RT), and was attributed to *f*-electron delocalization^3^. Similar studies were performed on the Ce_70_Al_10_Ni_10_Cu_10_ glass and again attributed to the *γ*-*α* transition in crystalline Ce due to *f*-electron delocalization^4^. These structural changes were reversible and the glasses recovered the original structure when the pressure was removed. For the Pd-based glasses changes in the structure under pressure at room temperature (RT) were continuous and reversible^5^. The pair distribution function (PDF) could be scaled with pressure and thus it was deemed that glass did not go through any structural transition.

There are reports of possible structural change in the Zr and La based metallic liquid state upon heating^6, 7^ at temperatures ~2*Tg* and at ~1.2 *Tg* ^8^. The possibility of such a liquid-to-liquid (L-L) transition has been vigorously pursued both by simulations and experiments. Published reports indicate a first-order L-L phase change in liquid phosphorous^9^ under pressure and water-glycerol mixture^10^ (see also ref. 11), and Al_2_O_3_-Y_2_O_3_^12^. Also there are rich literatures on pressure effects in inorganic and biological systems^13^.

Structural changes under pressure are usually studied by x-ray diffraction using a diamond anvil cell to generate pressure, but the geometry of the cell severely limits the available range of *Q*, the momentum transfer in diffraction. The structure of liquid and glass is described using the pair distribution function which requires high quality structure function, *S*(*Q*), determined over wide *Q* space^14^. In addition it is difficult to subtract from the data the background arising from the diamond cell due to diffuse scattering, which depends also on pressure and cell orientation.

We quenched supercooled metallic liquids under pressure to the glassy state and studied their structure by high-energy x-ray diffraction. By this approach we captured the structure of metallic liquid under pressure because the quenched glass should retain the basic structure of the liquid before quench. We discovered that the structure of a Zr-based metallic glass quenched from the supercooled liquid state under high pressure is different from the glass quenched without applied pressure. The structure, albeit stable at RT, reverted to the original one after annealing without pressure. This is the first experimental observation of such polyamorhpic, stable at ambient conditions and reversible by annealing, glass structure obtained by quenching under pressure from the Zr-based supercooled metallic liquid.

## Results and Discussion

### X-ray diffraction and PDF

In this work we brought Zr-based metallic glasses, Zr_50_Cu_40_Al_10_ (Alloy A) and Zr_50_Cu_47_Al_10_Pd_3_ (Alloy B), to the supercooled liquid (SCL) region under high pressure, and quenched them under pressure to room temperature. We denote these samples as High Pressure Quenched (HPQ) glasses. These glasses are stable after decompression at RT. The HPQ samples were characterized at ambient conditions using high-energy x-ray diffraction which produced high quality structure functions, *S*(*Q*), up to 23 Ǻ^−1^. Consequently, by Fourier transformation, reliable pair-density functions (PDFs) with high resolution in real space were obtained.

**Figure 1:**
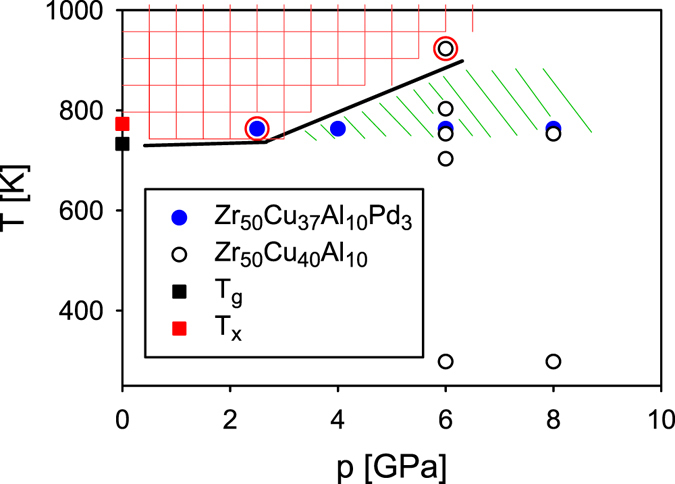
The T-P range explored by quenching the supercooled liquid state. Red circle indicates a nanocrystalline state. Black and red squares denote the glass transition and crystallization temperatures measured during heating at ambient pressure in the DSC for the as cast sample. The red area above the black line indicates where crystallization occurs. The green area indicates the range where the high-pressure structure was observed.

Figure 1 summarizes our experimental pressure-temperature ranges and the final sample state after quenching from the SCL as determined by X-ray diffraction and DSC. The red circle around the point indicates that the HPQ sample is in a crystallized state. Evidently application of some minimum pressure is needed to suppress crystallization in the supercooled liquid, ~3 GPa, and HPQ samples are glassy if higher pressure is applied. Such retardation of crystallization under high pressure was observed in several studies before^15, 16^. It should be noted that in our experiment samples are compressed at RT and then heated under pressure. As evident from Fig. 1, applying pressure after heating the sample to the SCL state would result in crystallization. Thus the only way to reach the transition is to apply high enough pressure (>3 GPa) first and then to heat up. The data points at ambient pressure were determined by DSC with the heating rate of 20 K/min. Supplementary Figure S1 shows DSC scans of sample A (quenched from 6 GPa and 803 K) and sample B (8 GPa and 763 K).

**Figure 2:**
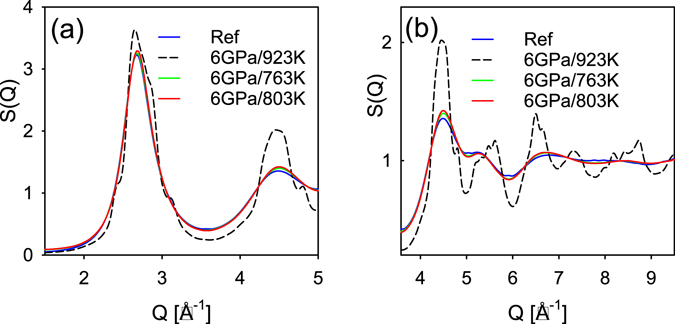
(**a**,**b**) The structure functions for several HPQ samples of Glass A (Zr_50_Cu_40_Al_10_) shown in two Q ranges. Panel (a) shows small change in the main peak for various glassy samples. Panel (b) shows significant changes in the higher order peaks and troughs, indicating changes in the atomic short range order. The 923 K/6 GPa sample is clearly nano-crystalline.

The structure functions for several samples of Glass A are shown in Fig. 2(a) and (b). Figure 2(a) presents *S*(*Q*) in the range of the first peak. Figure 2(b) shows the higher order peaks of *S*(*Q*). The main peak position of the *S*(*Q*) remains largely unchanged at 2.681 (±0.010) Å^-1^ with small changes in shape. However, the higher order peaks in (b) exhibit clear shifts and changes in amplitude, indicating a change in the short range atomic structure induced by high pressure in the supercooled region. The sample quenched from 923 K under 6 GPa is clearly nano-crystalline. This conclusion is obvious by inspecting the shapes of the main and the higher order peaks. However, this sample could be mistaken for a glass by low resolution diffraction measurement.

**Figure 3:**
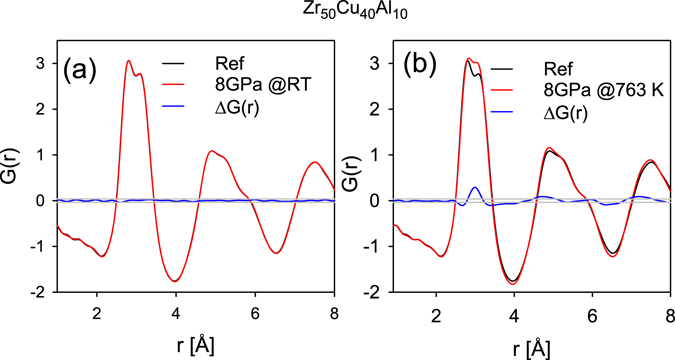
(**a**,**b**) *G*(*r*)’s for the glass that was (**a**) compressed to 8 GPa at RT and then decompressed and (**b**) quenched from 763 K at 8 GPa and then decompressed at RT. The difference in *G*(*r*) from the reference sample is plotted in blue line. Thin gray band denotes two standard deviations in statistical error.

Figure 3a shows the reduced pair distribution function *G*(*r*) for Glass A determined after compression to 8 GPa and decompression at room temperature, compared to *G*(*r*) of the reference (as-cast) sample. We also plot the difference between the two *G*(*r*)’s and statistical error bands, ±σ. The difference in *G*(*r*) in Fig. 3a is small and within error bars. This difference also defines our systematic experimental error due to x-ray beam-line optics, detector, and sample background uncertainties. We note that the slopes of *G*(*r*) for these two samples at small *r* up to the first peak trace each other quite well, indicating consistent data reduction and normalization. It is seen that compression to 8 GPa at RT followed by decompression does not affect the structure and the glassy sample recovers its original state.

However, the HPQ samples significantly differ in structure. Figure 3b presents *G*(*r*)’s for the glass that was quenched from 763 K at 8 GPa, and the difference in *G*(*r*) compared to the reference sample. The change in the structure induced by HPQ shown here is clearly beyond statistical and systematic errors. The second subpeak of the main peak becomes higher and shifts slightly towards smaller *r*. In addition, all peaks of *G*(*r*) become narrower, and consequently the amplitudes of oscillation become larger. Thus there is a clear change in the short-range order (first PDF peak) and consequently in the medium range order. However, there is no change in the apparent coordination number *N*(*r*) = 14.0 ± 0.2. Here *N*(*r*) was obtained by integration of 4*πr* ^2^ *ρ*(*r*) from 2.1 to 3.8 Å, using the average atomic density *ρ*_0_ = 0.054 atoms/Å^3^.

**Figure 4:**
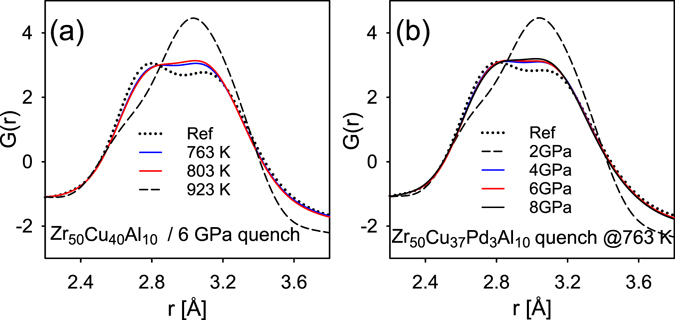
(**a**,**b**) Panel (a) shows the first PDF peak for Glass A quenched from the SCL under the same pressure 6 GPa from different temperatures and panel (b) first PDF peak for glass B quenched from the SCL from the same temperature 763 K but at various pressures.

Figure 4a shows the first PDF peak for Glass A quenched from the SCL under the same pressure 6 GPa from different temperatures, and Fig. 4b for glass B quenched from the SCL from the same temperature 763 K but at various pressures. The changes in the first peak of *G*(*r*) after HPQ are not gradual but stepwise, and are also different from those due to crystallization (dashed line). Once the transition occurred in the SCL the increasing pressure or temperature only make small additional changes.

### Issues with relaxation and crystallization

**Figure 5:**
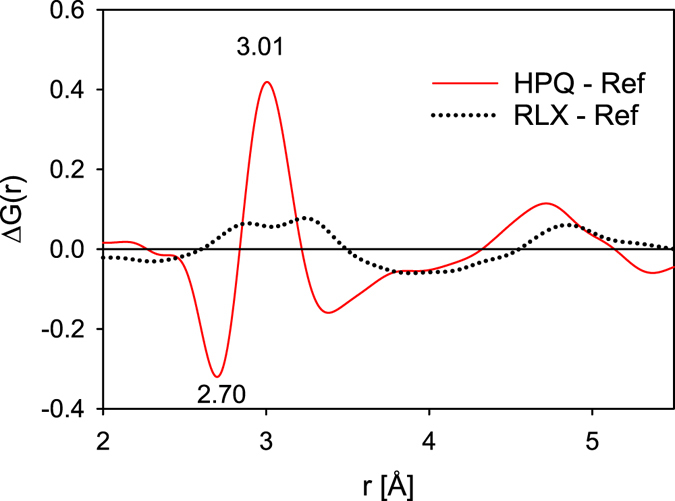
The difference *ΔG*(*r*) after HPQ and after structural relaxation, *ΔG*_*SR*_(*r*). The *ΔG*(*r*) is the difference between HPQ and the as cast sample and the *ΔG*_*SR*_(*r*) is the difference in *G*(*r*) between the sample annealed at *T*_*g*_ and the as cast sample.

In Fig. 5 we compare the difference *ΔG*(*r*) after HPQ to the change induced by structural relaxation, *ΔG*_*SR*_(*r*). Here *ΔG*_*SR*_(*r*) is the difference in *G*(*r*) between the reference (as-cast sample) and the sample annealed at *T*_*g*_. It is known that structural relaxation leads to small increase in density because of relaxation in the atomic-level stresses^17, 18^. This comparison shows that the changes in the structure due to HPQ from the SCL state are not arising from structural relaxation. The difference in the *G*(*r*) for the HPQ samples is qualitatively different and much more substantial. The amplitude in Fig. 5 is about 0.4, which is about 10% of amplitude in *G*(*r*) (Fig. 3a,b). The area under the difference in Fig. 5 |Δ*G*(*r*)|, is about 9% of the |*G*(*r*)| (in the range 2.0–4.0 Å).

**Figure 6:**
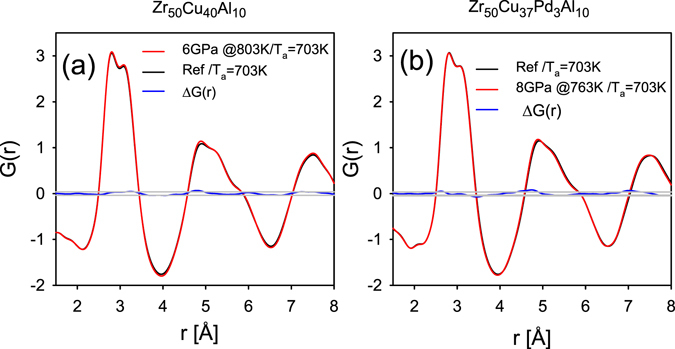
(**a**,**b**) The *G*(*r*) for the samples that were quenched under high pressure from the SCL, and then annealed at ambient pressure 5 degrees below the glass transition temperature. The reference (as cast) samples were also annealed at the same temperature for the comparison.

To eliminate the possibility that the change is due to nanocrystallization, we checked reversibility. Figure 6a,b shows the *G*(*r*) for the samples that were quenched under high pressure from the SCL, and then annealed at ambient pressure 5 degrees below the glass transition temperature for 5 mins. Because theses samples were annealed below *T*_*g*_, the result should be compared to the as-cast sample annealed for 5 mins at the same temperature (*T*_*g*_ − 5) as shown in Fig. 6. The difference in *G*(*r*) is very small and the remaining residue is due to the residual structural relaxation and noise. Thus this comparison shows that the original (ambient pressure) structure is substantially recovered. These results confirm that the structural changes induced by pressure in the SCL were not caused by structural relaxation or crystallization.

### Computer simulation

**Figure 7:**
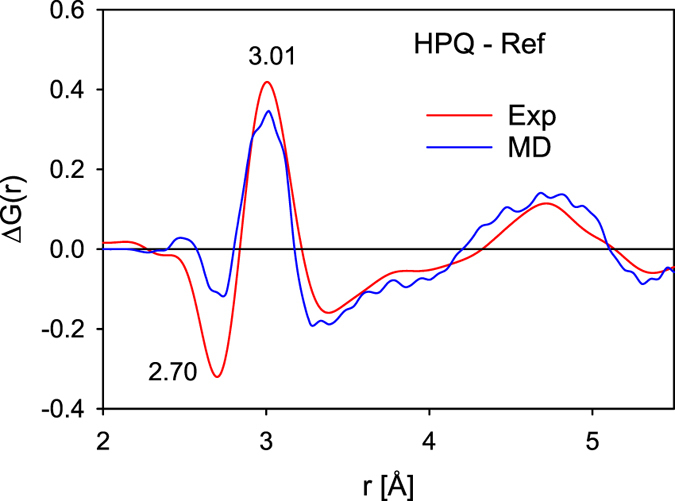
The difference in *G*(*r*) compared to the as cast sample, Δ*G*(*r*), for the experimental HPQ sample and the HPQ obtained by MD simulation.

We have also performed molecular dynamics (MD) simulation of the HPQ process. The reference sample was hydrostatically compressed to 10 GPa at 300 K, heated up under 10 GPa to 1000 K, and then cooled down under pressure to 300 K and decompressed. Because *T*_*g*_ of the model (~850 K) is higher than the experimental *T*_*g*_, a higher treatment temperature was chosen. The pair distribution functions were weighted by atomic numbers and atomic concentrations to compare with the X-ray PDF. Figure 7 shows the difference in *G*(*r*) compared to the reference sample, Δ*G*(*r*), for the experimental HPQ sample and the HPQ obtained by MD simulation. Qualitatively the changes observed in the *G*(*r*) by MD simulation are similar to the experimental results, especially at distances where Zr-Zr pairs contribute (2.9 Å and above). Examination of the partial Zr-Zr PDF obtained in MD confirms the change in the distribution and smaller average separation of Zr-Zr distances (Supplemental Figure S3). At the same time the Cu-Zr peak shifts slightly to the right, which may be an indication of changes in the short-range order of Cu because of Zr rearrangements. The most frequently found Voronoi polyhedra centered on Cu, Al and Zr in the reference, HPQ and high temperature samples are shown in Supplemental Figure S4a–c. The results show that the icosahedral clusters centered on Al and Cu are more frequently seen after applying pressure in the liquid state, however local polyhedral packing changes rather continuously with pressure (Figure S5a–c). The MD results are consistent with the idea that hydrostatic pressure in the SCL region leads to increased ordering and a new state with higher density (better packing).

### Nature of the structural change

The change in the structure with pressure (see Fig. 4) is not gradual but stepwise, occurring in the range below 4 GPa which is not accessible because of crystallization. For this reason we conclude that *a phase change in the supercooled liquid state occurred*, at a pressure between 0 and 4 GPa. However, we cannot determine the precise value of the critical pressure because at ~3 GPa and below SCL crystalizes. Also the isothermal compression of the SCL starting from ambient pressure is not viable because of the crystallization. Figure 1 shows the area where the high-pressure structure was observed by green shade. Note that this is not a phase diagram. The real phase boundary must be nearly vertical, and between 0 and 4 GPa, and the high-pressure phase should exist to the right of this line. However, it cannot be seen at high temperatures because of crystallization, limited by the black line in Fig. 1, and also at low temperatures because of glass transition (a nearly horizontal line near 720 K) because of kinetic slowdown. Consequently the high-pressure phase can be observed only in a very limited range of temperature and pressure, indicated by green shade.

The main structural change due to this transition is the asymmetric shift of the right side shoulder of the first peak representing Zr-Zr pairs in *G*(*r*) as is seen in Fig. 4. It is observed that positive change is centered at about ~3 Å whereas it is negative below 2.84 and beyond 3.2 Å (Fig. 5). Thus there is a change in the distribution of atomic distances so that those in the right shoulder of the first peak in *G*(*r*) are moving towards the center due to pressure. The main contribution to the *G*(*r*) comes from the Cu-Zr and Zr-Zr partial PDFs (because Al and Cu scatter x-ray more weakly than Zr) with the expected average distances at ~2.75 and 3.12 Å in the reference sample. Because the right shoulder represents Zr-Zr pairs this implies that some of the long Zr-Zr pairs become shorter, thus the average distance and the spread become smaller, by HPQ. In the sample cooled without pressure the Zr shells centered on Zr are more distorted, whereas high pressure drives these shells more symmetric, reducing distortion. This conclusion is corroborated by a comparison of two binary alloys Zr_50_Cu_50_ and Zr_35_Cu_65_ shown in Supplementary Figure S3a,b. The change in the structure due to high pressure quenching from the supercooled liquid state is found to be significant in the Zr_50_Cu_50_ alloy but negligibly small in the Cu-rich Zr_35_Cu_65_ alloy.

Our results demonstrate that when the sample is quenched without pressure the Zr environment centered on Zr atom is significantly distorted with short and long Zr-Zr distances. Such distortions are either removed or reduced by pressure, and a distinct structure is obtained. We speculate that this transition reflects the unique nature of Zr-based metallic glasses in which Zr covalency competes against the tendency of close packing. Zr has partially filled *d*-electron orbitals, with the electron density^19^ around 3. Therefore Zr-Zr bonding has a significant covalent character, as suggested by their low fragility^20^. In particular, the Zr-Zr *d-d* bonding prefers distorted Zr environment (discussed further in Supplementary Information) resulting in Zr-Zr bonds split into short and long bonds. However, such a structure is not tightly packed. Upon applying high pressure in the liquid state the principle of packing into the dense-random-packed structure overcomes covalent bonds, and more highly symmetric local structures with higher density are produced. This is analogous to the transition induced by pressure from a low-density amorphous state dominated by covalent bonding to the high-density amorphous state preferred by packing, which are seen in silicon^21^, germanium^22^ and ice^23^.

The polyamorphic transition in Ce-based glass occurs because Ce has an electronic transition in the *f*-shell and under pressure its size becomes smaller. However, after pressure is removed the original structure is recovered – because the electronic transition in Ce can be stabilized only by pressure. High pressure quenched (HPQ) samples are quenched from high temperature supercooled liquid under pressure, and are stable in ambient condition i.e. at RT and ambient pressure. The HPQ samples have a different structure and mirror the pressure induced structure in the liquid state.

In this contribution we clearly show that: HPQ samples are stable at ambient condition and they have a different structure than glasses quenched without applied pressure (Fig. 2b); the change in the HPQ structure is not due to structural relaxation, which is a standard aging process for glasses (Fig. 4a); HPQ sample transforms back to the structure quenched without pressure, which proves that pressure induced change is not caused by nano-crystallization (Fig. 4b).

The data provides indirect evidence that pressure induces a phase transition in the supercooled liquid. The pair distribution function shows that without pressure the Zr neighbor shell of Zr is distorted, whereas such distortion is removed by applying pressure in the liquid state and the structure becomes better packed. The results indicate that Zr-based alloy liquid has two distinct structures, a low-density more distorted structure at ambient pressure, and a high-density better packed structure under pressure. The Zr-shell distortion in metallic glasses formed under ambient pressure reflects covalency of Zr-Zr bonding. Quenching under pressure suppresses such distortion and HPQ glasses have more densely packed structure.

## Methods

### Samples

The preparation of Zr_50_Cu_40−*x*_Al_10_Pd_*x*_, (*x* = 0, 3) metallic glass samples is described in detail in ref. ^24^. The glass transition temperature (708 K) and crystallization temperature (773 K) were determined by calorimetric measurements (DSC-Perkin Elmer 7) with a heating rate of 0.33 K/sec (20 deg/min). Both glasses have similar glass and crystallization temperatures. Glassy structure was confirmed by a high energy X-ray diffraction in transmission geometry. The reference sample is in the as-cast state.

### High Pressure Quenching

Cylindrical shaped samples with a height ~4 mm were used in the high pressure quench (HPQ) experiments. The details of the HPQ setup is described elsewhere^25^. The sample was inserted into a graphite tube that acted as a heater and was padded with boron nitride as filler. The pressure and temperature were calibrated using standard compounds and the thermocouple respectively. The temperature has uncertainty of ±25 deg. Each sample was compressed at RT to the final pressure and heated to reach the target temperature, held for about 3 min, and then cooled down to room temperature. At room temperature samples were decompressed and removed from the HP cell. The approximate cooling rate in the HP cell is ~5–10 K/min.

### Structural studies and data analysis

High-energy x-ray scattering experiments were performed at the beamline P07, Desy, Hamburg, and 1-ID of the Advanced Photon Source, Argonne National Laboratory. The incident energy was 100 keV, the beam size was 0.2 × 0.2 mm^2^, and a 2 dimensional (2D) stationary detector was placed ~40 cm away from the sample. The samples had comparable thickness, (0.55–0.63 mm) resulting in negligible systematic errors due to background and absorption corrections. The collected intensities had been normalized to incident beam monitor recorded by an ion chamber in front of the sample. Azimuthally integrated intensities were processed using the pdfgetX2^26^ package to obtain the structure function, *S*(*Q*), where *Q* = 4*π*sin *θ*/*λ*, *θ* is the diffraction angle and *λ* is the x-ray wavelength, up to ~23 Ǻ^−1^. In the process, background corrected for sample absorption had been subtracted. Multiple scattering and Compton scattering were properly removed and data had been normalized to absolute electron units. Then *S*(*Q*) was Fourier-transformed to obtain the reduced pair distribution function, *G*(*r*), by

1 $$G(r)=4\pi r(\rho (r)-{\rho }_{0})=\frac{2}{\pi }\int Q[S(Q)-1]\sin (Qr)dQ$$

where *ρ*(*r*) is the pair density function (PDF) and *ρ*_0_ is the atomic density^10^. Propagation of error in the PDF analysis is described in ref. 27.

### Molecular Dynamics

Molecular dynamics simulations were performed for a ternary Zr_50_Cu_40_Al_10_ alloy. We used LAMMPS^28^ with the EAM potentials generated by H. W. Sheng^29^ for ZrCuAl. Sample size was 32000 atoms, initially placed randomly in the fcc lattice. Sample was melted at 2500 K and then quenched to 300 K under the NPT assemble. For each pressure and temperature sample was equilibrated for 1 ns. Temperature was gradually decreased by 50 deg in each step. Hydrostatic pressure was applied in 1 GPa steps. Voronoi polyhedral analysis was performed using Voro++ library^30^. The glass transition in MD sample is ~850 K.

### Data availability

The data is available in form of Excel files from wdmowski@utk.edu on e-mail request.

## Electronic supplementary material

Supplemental Information
