# Supplementary material for: Pressure Induced Liquid-to-Liquid Transition in Zr-based Supercooled Melts and Pressure Quenched Glasses
Source: Sci Rep. 2017 Jul 26;7:6564. doi: 10.1038/s41598-017-06890-w (PMC5529562; doi:10.1038/s41598-017-06890-w)
Supplement: Supplementary file 1 — Supplemental Information [file 41598_2017_6890_MOESM1_ESM.pdf]

Supplemental Information to "Pressure Induced Liquid-to-Liquid Transition in Zr-based Supercooled Melts and Pressure Quenched Glasses"

W. Dmowski, S. Gierlotka, Z. Wang, Y. Yokoyama, P. Palosz and T. Egami

1. Results of Differential Scanning Calorimetry

The High Pressure Quenched (HPQ) samples, ~ 20 mg in weight, were examined by the differential scanning calorimetry (DSC). The scans displayed in the Figure S1 show that HPQ samples have DSC trace typical for Zr-based metallic glasses. They exhibit glass transition temperature T_g, followed by super-cooled region and finally crystallization peak. The difference in the glass temperature between two compositions is negligible with this heating rate. As is discussed in the main text the HPQ glasses relax quickly approaching SCL.

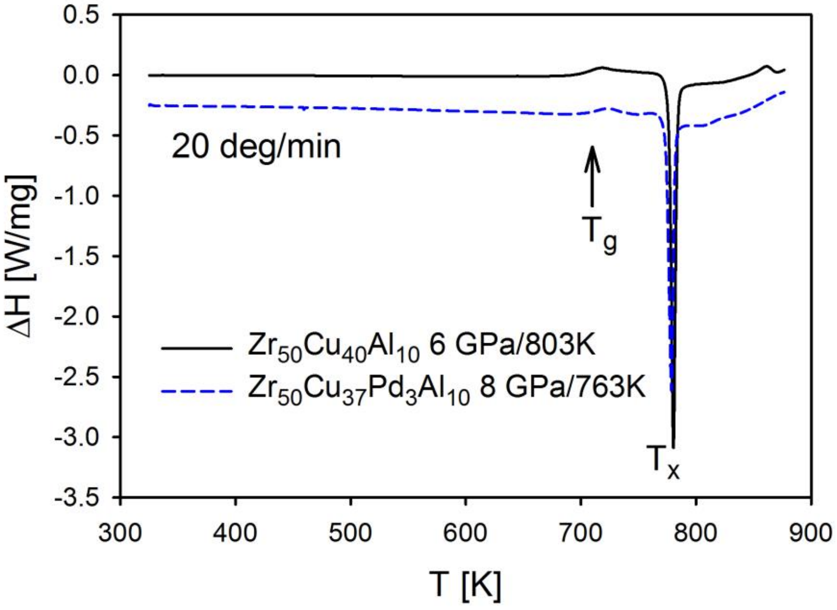

Fig. S1. DSC scans for the HPQ samples.

2. Composition Dependence

Figure S2a-b compares structure after high pressure annealing in the SCL region for two binary alloys $Zr_{35}Cu_{65}$ and $Zr_{50}Cu_{50}$. The G(r) is dominated by two major contributions from Cu-Zr (left side of the first peak) and Zr-Zr (right side of the first peak) atomic pairs. It is evident that

high concentration of Zr is needed to induce significant structural change under high pressure in the SCL region. The reference samples are in the as cast state.

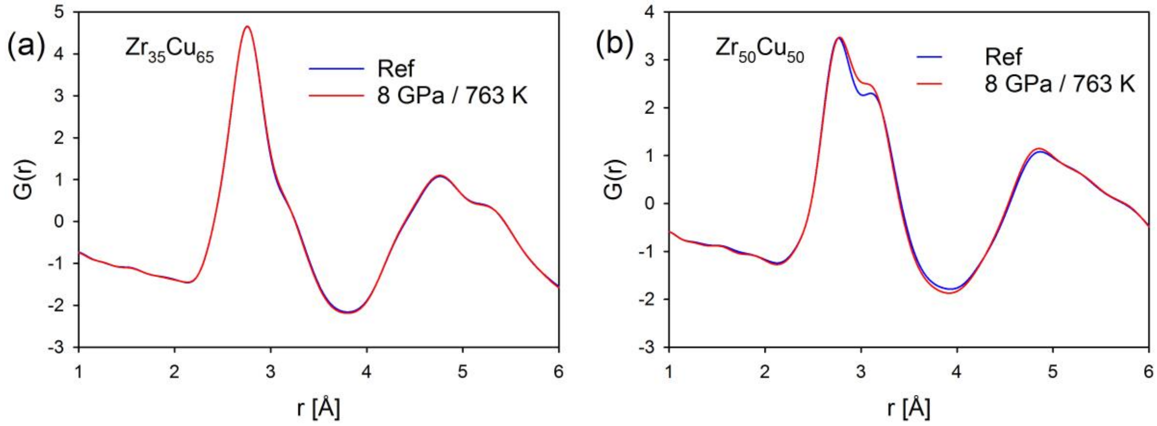

Figure S2a-b. Comparison of the G(r) of the HPQ samples obtained by quenching high pressure from the indicated temperature in the SCL region with the reference samples obtained by quenching under ambient pressure.

3. Partial PDF from MD

Figure S3 shows partial pair distribution functions obtained from the MD structures that contribute most to the total PDF. The HPQ sample is quenched under 10 GPa from 1000 K. The plot shows contraction in the average distance between Zr-Zr pairs indicating change in the Zr distribution in the alloy. At the same time Zr-Cu peak becomes lower and slightly shifted due to changed coordination of Cu by Zr atoms. Overall contribution from the Cu-Cu pairs is small.

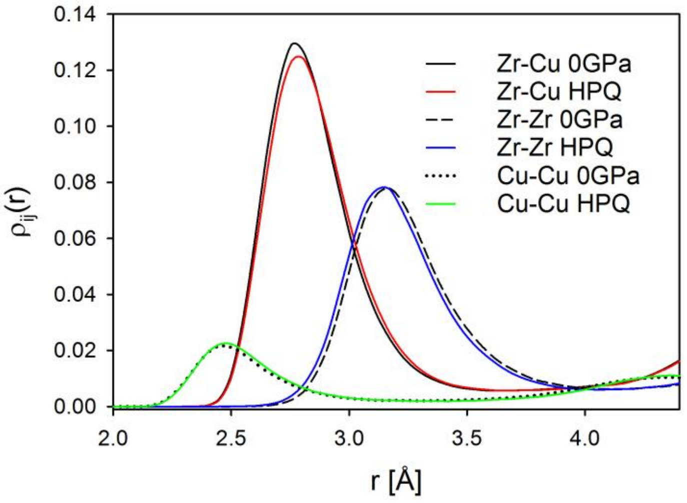

Figure S3. Partial PDFs obtained from the 32,000 atoms sample in MD simulations. HPQ samples are quenched under 10 GPa from 1000 K.

4. Local Structure

Structures obtained by MD simulations were used in the analysis of most frequent polyhedra arrangements in the samples. Figures S4a-c show Zr, Cu and Al centered polyhedra that occur with frequency higher than 2% threshold. The four bars compare data at room temperature for the MD sample quenched under zero pressure, HPQ sample, and two sets at 1000K: at 0 and at 10 GPa. Zr-centered polyhedral distribution does not seem to change considerably as a result of pressure. The effect of the pressure is to mainly increase the height of the bar indicating increasing ordering and better packing in the system. On the other hand Cu- and Al-centered polyhedra show progressive increase in frequency of icosahedral ordering induced by pressure. The same trend is observed in simulation at 1000 K in liquid state at different pressures as seen in Figure S5a-c.

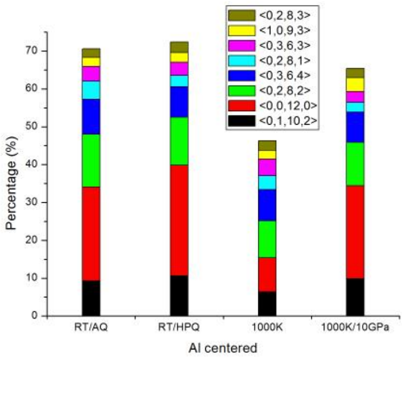

Figure S4a. Al-centered.

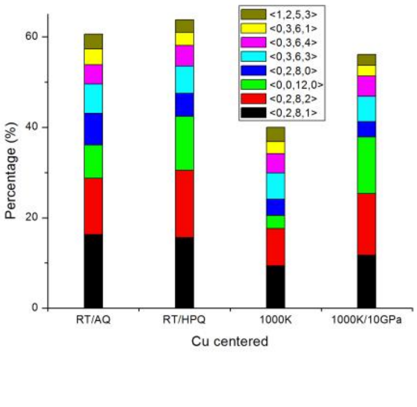

Figure S4b. Cu-centered.

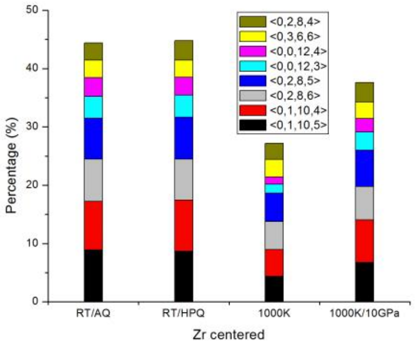

Figure S4c. Zr-centered.

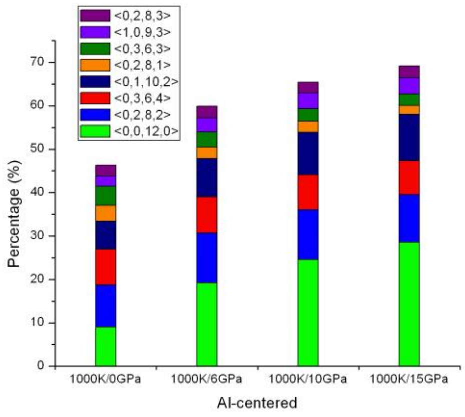

Figure S5a. Al-centered.

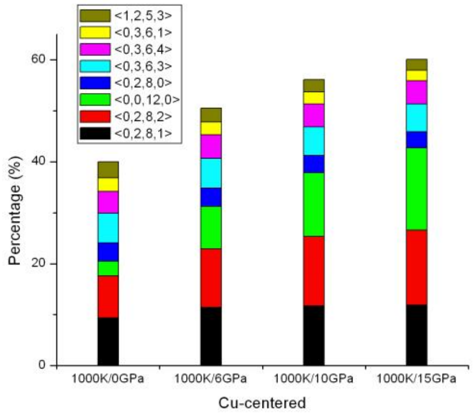

Figure S5b. Cu-centered.

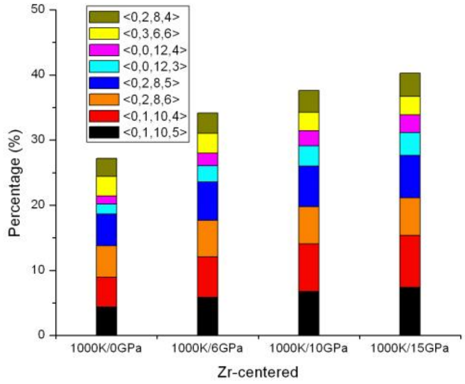

Figure S5c. Zr-centered.

5. Zr covalency.

In a cubic crystal the crystal-field or ligand field splits the *d*-electron states into doubly degenerated e_g state and triply degenerated t_{2g} state [1]. Even in glasses and liquids such symmetry components lifts the degeneracy of the *d*-state. Because Zr has about 3 *d*-electrons they are likely to occupy the e_g-like states, leaving t_{2g}-like states open. In cubic crystals the e_g state produces 6 bonds and t_{2g} 8 bonds. In glasses among the 12 – 15 neighbors of Zr only up to 6 are covalently bonded and short, leaving other bonds longer (non-bonding or metallic bonding). This explains why Zr-Zr bonds split to short and long bonds. Upon application of pressure packing requirement makes the environment of Zr more spherical, mixing e_g and t_{2g} states. Thus under pressure Zr-Zr bonds are less covalent, but better packing is achieved.

Supplement References

1. A. R. West, Solid state chemistry and its applications, John Wiley and Sons, 1980.
